# Domestic

**Published:** 1841-09-18

**Authors:** 


### DOMESTIC.

### HEALTH OF THE CITY

INTERMENTS *in the City and Liberties of Philadelphia, from the 4th to the 11th of September.*

| Diseases. | Adults. | Children. |
|---|---|---|
| Apoplexy, | 1 | 0 |
| Bowel Complaint, | 0 | 14 |
| Biliary Calculi, | 1 | 0 |
| Croup, | 0 | 1 |
| Consumption of the lungs, | 0 | 4 |
| Convulsions, | 1 | 1 |
| Diarrhœa, | 2 | 3 |
| Dropsy, | 2 | 1 |
| —— of the head, | 0 | 6 |
| —— Breast, | 1 | 0 |
| Disease of the brain, | 0 | 1 |
| Drowned, | 0 | 1 |
| Dysentery, | 2 | 0 |
| Debility | 0 | 4 |
| Epilepsy, | 0 | 1 |
| Enlargement of heart, | 2 | 0 |
| Effusion on brain, | 0 | 1 |
| Fever, Remittent, | 1 | 0 |
| —— Typhus, | 0 | 1 |
| —— Puerperal, | 2 | 0 |
| Hæmorrhage, | 1 | 0 |
| Inflammation of the Brain, | 0 | 5 |
| —— Bronchi, | 1 | 1 |
| —— Lungs, | 2 | 1 |
| —— Stomach, | 0 | 1 |
| —— Stomach and Bowels, | 0 | 1 |
| —— Bowels, | 2 | 0 |
| —— Breast, | 0 | 1 |
| | — | — |
| *Carried forward,* | 27 | 49 |

| Diseases. | Adults. | Children. |
|---|---|---|
| *Brought forward,* | 27 | 49 |
| Intussusception, | 1 | 0 |
| Inanition, | 0 | 1 |
| Jaundice, | 0 | 1 |
| Marasmus, | 0 | 4 |
| Measles, | 0 | 1 |
| Mortification, | 1 | 0 |
| —— of Arm, | 1 | 0 |
| Palsy, | 1 | 0 |
| Rupia, | 0 | 1 |
| Small pox, | 2 | 4 |
| Still-born, | 0 | 6 |
| Teething, | 0 | 2 |
| Unknown, | 0 | 2 |
| | — | — |
| Total, 104— | 33 | 71 |

Of the above, there

| | |
|---|---|
| were under 1 year, | 39 |
| From 1 to 2 | 18 |
| 2 to 5 | 7 |
| 5 to 10 | 2 |
| 10 to 15 | 1 |
| 15 to 20 | 4 |
| 20 to 30 | 5 |
| 30 to 40 | 9 |
| 40 to 50 | 6 |
| 50 to 60 | 1 |
| 60 to 70 | 2 |
| 70 to 80 | 9 |
| 80 to 90 | 1 |
| Total, | 104 |

Of the above there were 4 from the almshouse, 5 people of colour, and 1 from the country, which are included in the total amount.

